# Research on Heterogeneous Traveler Travel Mode Choices with Differences under a Mixed Traffic Environment

**DOI:** 10.3390/s23136091

**Published:** 2023-07-02

**Authors:** Yutong Shen, Yuelong Wu, Baozhen Yao

**Affiliations:** 1 School of Transportation Science and Engineering, Beihang University, Beijing 100191, China; 2 State Key Laboratory of Structural Analysis for Industrial Equipment, School of Automotive Engineering, Dalian University of Technology, Dalian 116024, China

**Keywords:** shared automated vehicle, multi-mode choice, heterogeneous travelers, travel utility

## Abstract

Autonomous vehicles (AVs) have been made possible by advances in sensing and computing technologies. However, the high cost of AVs makes privatization take longer. Therefore, companies with autonomous vehicles can develop shared autonomous vehicle (SAV) projects. AVs with a high level of automation require high upgrade and use costs. In order to meet the needs of more customers and reduce the investment cost of the company, SAVs with different levels of automation may coexist for a long time. Faced with multiple travel modes (autonomous cars with different levels of automation, private cars, and buses), travelers’ travel mode choices are worth studying. To further differentiate the types of travelers, this paper defines high-income travelers and low-income travelers. The difference between these two types of travelers is whether they have a private car. The differences in time value and willingness to pay of the two types of travelers are considered. Based on the above considerations, this paper establishes a multi-modal selection model with the goal of maximizing the total utility of all travelers and uses the imperial competition algorithm to solve it. The results show that low-income travelers are more likely to choose buses and autonomous vehicles with lower levels of automation, while high-income travelers tend to choose higher levels of automation due to their high value of travel time.

## 1. Introduction

With the development of sensing and computing technologies, autonomous vehicles (AVs) have been widely studied and applied in some areas [1,2,3]. However, due to their high requirements for sensing and computing equipment, the cost is much higher than that of ordinary private vehicles (PVs) [4,5]. Therefore, AVs are not privately owned at present and are only owned by some large enterprises. Shared autonomous vehicles (SAVs) have become a way for people to adapt and further accept AVs where the ownership of AVs belongs to the enterprise and users pay user fees [6]. Compared with PVs, the advantage of SAVs is that it does not require users to drive the vehicle and can automatically find parking spaces for parking, thereby freeing users’ hands and improving their user experience [7]. Therefore, the driver’s travel time and the value of travel time (VOTT) can be greatly reduced [8]. Compared with buses, SAVs are more flexible and comfortable. These advantages make SAVs competitive. Hence, in the case of the coexistence of PVs, buses, and SAVs, people have more choices when traveling, and each of these modes of transportation has its own advantages and disadvantages. Therefore, people’s choice of travel mode is worthy of in-depth research and discussion.

Levin and Boyles [9] studied the mode choices of different classes of travelers in the case of the coexistence of AV travel, PV travel, bus travel, walking, and bicycling. In their research, AV could autonomously find parking spaces and increase link capacity, which not only helped AV users reduce the time to find parking spaces but also improved the travel efficiency of other travelers. Therefore, with the increase in AVs on the road, the congestion of road traffic is alleviated. Both the travel mode and the choice of workplace were considered in Childress et al.’s study [10]. The test results showed that high-income people were more willing to choose AV travel. Because the VOTT of high-income people is higher, and AVs can allow them to use the time in the vehicle to work, it reduces their in-vehicle time VOTT. Chen and Kockelman [11] studied the traveler’s mode selection based on the Logit model, among which the travel modes included PVs, transit, and shared autonomous electric vehicles.

Yao et al. [12] conducted a survey on the selection preferences of SAV and analyzed their potential user characteristics. They classified historical travel modes based on the k-means clustering method and used factor analysis to classify personality and attitude characteristics. A mixed logit model was established for two different populations, with two explanatory variable parameters affected by different distributions. The results showed that the characteristics of travel modes had an extremely significant impact on travelers’ mode selection behavior. Personality and attitude traits are important factors that influence a traveler’s choice of SAV, and their significance is significantly higher than the importance of socio-economic attributes, such as gender and age. Malokin et al. [13] studied the coexistence of the commuter railway, public transport, private cars, and autonomous vehicles and proposed a preference mode selection model, which explained the impact of multi-task attitude and behavior on the effectiveness of various alternatives. The model estimated that if there is no option to use a laptop or tablet computer when commuting, the shares of commuter railways, buses, and private cars will decrease by 0.11, 0.23, and 1.18 percentage points, respectively. On the contrary, in the hypothetical scenario of autonomous vehicles, cars will be allowed to participate in production activities, and the share of driving alone will increase by 48 percentage points.

However, heterogeneous travelers, named high-income travelers and low-income travelers, and SAVs with different levels of automation, have not been considered. High- and low-income travelers have large differences in terms of time value cost, willingness to pay for travel costs, and acceptance of public transport congestion. Therefore, in the case of limited travel modes and travel capacity constraints, the competition of travelers with different incomes needs to be considered in depth. The improvement of the automation level of SAVs necessitates high costs, and higher automation levels of SAVs require higher usage costs. To attract more travelers to choose SAVs, SAV operators need to provide as many types of SAVs as possible, which are then chosen by travelers with different conditions or characteristics. Therefore, the attractiveness of SAVs with different levels of automation to travelers with different income levels needs to be considered. We consider three levels of automation for SAVs, with the L0 SAVs having the lowest level of automation, requiring users to be prepared to take over the driving rights at all times. L1 SAVs require users to take over the driving rights in extreme situations. L2 SAVs have the highest level of automation and do not require user intervention throughout the entire process. Based on the above considerations, this paper establishes a multi-modal selection model based on heterogeneous travelers with the aim of maximizing the total utility of all travelers. This paper includes the following:

- Heterogeneous travelers, named high-income travelers and low-income travelers, are considered in this paper. The difference between these two types of travelers is whether they have a private car. High-income travelers have high VOTT and the ability to pay higher travel fees. Low-income travelers have low VOTT and are not sensitive to congestion and other situations. Therefore, based on the differences between the two types of travelers, a discussion of the differences in their choices of travel modes should be considered.
- SAVs with different levels of automation are considered. Because SAVs with high automation levels have high costs and can charge high user fees, SAV operators need time and finances to upgrade SAVs with different automation levels.
- In order to meet the needs of various travelers, SAVs with different levels of automation should be provided by operators, so the selection of SAVs with different levels of automation by heterogeneous travelers is worthy of further research.

The rest of the paper is arranged in this sequence: Section 2 summarizes the related research, including multiple mode selection models and the impact of SAVs on travel behavior. Section 3 introduces a mode choice model based on heterogeneous travelers. Numerical analysis is performed with the goal of maximizing the utility of all travelers in Section 4. Finally, the conclusion and future research directions are discussed in Section 5.

## 2. Related Work

In order to compare the contributions of different researchers to travelers’ travel mode choices, we established a comparison table to compare their contributions, as shown in Table 1. From the table, it can be seen that previous studies have conducted sufficient research on the selection of traveler travel modes, but few have studied SAVs with different levels of automation, and there is also limited research on the heterogeneity of travelers.

## 3. Materials and Methods

The society utility model presented by [3] is used to calculate the total utility of all travelers. The total utility calculation is shown in Section 3.1. Subsequently, the travel utility of different modes of travelers with different income levels is shown in Section 3.2.

### 3.1. The Calculation of Total Utility

To simplify the formulation of models, some notations are defined in advance. Two types of travelers, named high-income travelers and low-income travelers, are studied in this article. Let k∈K={1,2} represent a class of travelers, where k=1 represents high-income travelers, and k=2 denotes low-income travelers. To make it easier to distinguish between travelers with different income levels, we assume that travelers with PVs are high-income travelers. i∈M represents one travel mode where M is the set of all travel modes.

The total utility of all travelers can be calculated as follows:

(1) $$U(i,k)=\sum_{i=1}^{M}\sum_{k=1}^{K}X_{i}^{k}U_{i}^{k},\forall i\in M,k\in K$$

In Equation (1), $X_{i}^{k}$ and $U_{i}^{k}$ denote the number and utility of kth travelers traveling in mode i, respectively. $U_{i}^{k}$ includes two parts of fixed utility and variable utility, and is calculated as follows:

(2) $$U_{i}^{k}=-\alpha F_{i}-(1-\alpha )V_{i}^{k},\forall i\in M,k\in K$$

(3) $$F_{i}=\lambda_{i}D+C_{i},\forall i\in M$$

(4) $$V_{i}^{k}=-\psi (F_{i})\cdot VOTT_{i}^{k}\cdot \frac{D}{S}\cdot h_{i},\forall i\in M,k\in K$$

In Equation (2), $F_{i}$ denotes the fixed cost depending on the travel mode i, while $V_{i}^{k}$ indicates the variable utility relating to the travel mode i and the types of travelers k. α is the weight coefficient. $\lambda_{i}$ is the unit cost of the ith travel mode, and D is the distance. Therefore, $\lambda_{i}D$ is the distance-dependent variable cost of the ith travel mode. Let $C_{i}$ denote the fixed fees of the ith travel mode, such as the parking fees for PVs, the fare for buses, and so on. $\psi (F_{i})$ is a function that converts $F_{i}$ to time ($\psi (F_{i})=\eta_{k}\cdot F_{i}^{k^{\frac{3}{2}}}$ where $\eta_{k}$ is a parameter related to the types of travelers). $VOTT_{i}^{k}$ denotes the value of travel time of kth travelers traveling in mode i. S is the average velocity, so $\frac{D}{S}$ is the average travel time. $h_{i}$ denotes the degree of traveler discomfort. If the traveler chooses to travel by bus, $h_{i}$ represents the ratio of the total passengers on the bus to the capacity of the bus; if the traveler chooses PVs or SAVS, $h_{i}$ represents the degree of traffic congestion.

### 3.2. System Utility of Different Travel Modes

#### 3.2.1. The Utility of Travelers Traveling by PVs

As previously assumed, high-income travelers and low-income travelers are distinguished by whether they own a PV. The existence of PVs often leads high-income travelers to consider parking fees when driving PVs, which low-income travelers do not need to consider. Therefore, for the calculation of $C_{i}$, we need to further state that travelers who drive PVs need to pay the parking cost; that is, $C_{i}$ is the parking cost. The utility model of travelers traveling by PVs is:

(5) $$F_{i}=l_{i}D+C_{i},i=PV$$

(6) $$V_{i}^{k}=-\psi (F_{i})\cdot VOTT_{i}^{k}\cdot \frac{D}{S}\cdot h_{i},i=PV,\forall k\in K$$

#### 3.2.2. The Utility of Travelers Traveling by Buses

Different from PVs, travelers who choose public transportation usually take a considerable time to travel from their departure point to the bus stop and from the bus stop to their destination, which cannot be ignored. Therefore, the travel time of travelers who choose to travel by bus will consist of three parts, the time to reach the bus station, the travel time on the bus, and the time from the bus station to the destination. In general, the cost of traveling by bus is only a fixed bus fare and does not depend on the distance. So, here we use it as a basic assumption for the convenience of the following research. Therefore, the utility model of travelers traveling by bus can be described as:

(7) $$U_{i}^{k}=-\alpha C-(1-\alpha )V_{i}^{k},i=Bus,\forall k\in K$$

(8) $$V_{i}^{k}=-(\frac{VOTT_{i}^{k}}{60})\cdot \omega \cdot (T_{A}+T_{B}+T_{C})-VOTT_{i}^{k}(\frac{D}{S_{i}}),i=Bus,\forall k\in K$$

C is the bus fare. ω is the weight coefficient. $T_{A}$, $T_{B}$, and $T_{C}$ denote the time to reach the bus station, the travel time on the bus, and the time from the bus station to the destination, respectively.

#### 3.2.3. The Utility of Travelers Traveling by SAVs

Similar to the calculation of bus travel time, we assume that the traveler’s traveling time is also divided into three parts: $T_{A}$, $T_{B}$, and $T_{C}$, because the station of SAVs is a distance from both the departure point and the destination. However, the cost of a traveler traveling by SAVs will depend on the traveled distance, because the cost of SAVs is not fixed and constant, but it is positively correlated with the travel distance. In addition, we convert the upgrade costs of different levels of SAVs into customer usage fees. The utility of travelers traveling by SAVs is as follows:

(9) $$U_{i}^{k}=-a\left[l\times D+C\right]-(1-a)V_{i}^{k},i=SAV,\forall k\in K$$

(10) $$V_{i}^{k}=-(\frac{VOTT_{i}^{k}}{60})\cdot \omega \cdot (T_{A}+T_{B}+T_{C})-\varepsilon \cdot VOTT_{i}^{k}(\frac{D}{S_{i}})-Fare_{i}^{j},i=SAV,\forall k\in K$$

In Equation (11), ε indicates that the conversion coefficient $Fare_{i}^{j}$ can be calculated as follows:

(11) $$Fare_{i}^{j}=\left\{ \begin{array}{l} 0,j=0\\ \frac{A_{{}_{j-1,j}}\mu }{n\cdot \beta },j=1,2 \end{array}\right.$$

The 0th SAVs are defined as the benchmark; that is, the upgrade cost is based on it. $A_{{}_{j-1,j}}$ is the upgrade costs from the j−1th to the jth automation level of SAVs. n represents the total number of orders per year. β is the service life. μ denotes the profit factor.

## 4. Results

In this section, the multiple mode selection model with heterogeneous travelers is tested. The basic test results are shown in Section 4.1. Subsequently, the sensitivity based on the three parameters of the bus fare, the fuel cost per kilometer, and the SAV’s fee per kilometer are analyzed and discussed in Section 4.2 to further demonstrate the rationality of the model.

### 4.1. Basic Test Results

Some parameters are defined in advance. The parameters of heterogeneous travelers and the parameters of different travel modes are shown in Table 2 and Table 3, respectively. With the aim of maximizing the total utility of all travelers, the test results are shown in Table 4. From Table 4 we can see that 82.875% of the low-income travelers choose buses, while the rest of the low-income travelers choose the SAVs with the lowest automation level because low-income travelers are more sensitive to price and less concerned about time. For high-income travelers, 50% choose PVs, while the remaining 50% choose SAVs with the highest level of automation because high-income travelers are more sensitive to time and less concerned about price. From the results, we can also see that high-income people pay more attention to VOTT.

### 4.2. Sensitivity Analysis

As bus fare greatly affects traveler behavior, this parameter is analyzed and discussed in Section 4.2.1. With the change in fuel cost per kilometer and the SAV’s fee per kilometer, peoples’ choices will correspondingly change. The two factors play significant roles in changing peoples’ choices, so it is necessary to analyze the influence of the two factors. The two parameters are analyzed and discussed in Section 4.2.2 and Section 4.2.3, respectively.

#### 4.2.1. Sensitivity Analysis of Bus Fares

Buses are operated by the government. They are a public welfare mode of public travel. They have the advantages of lower prices and large capacities, and are widely selected by high- and low-income travelers. Therefore, this section studies the impact of bus fares on the travel mode choice of all travelers. We assume that the fare range of buses is 1 to 2 RMB. Under different fares, the number of travelers who choose different modes is shown in Table 5.

From Table 5, we can see that when the bus fare is 1 RMB, all low-income travelers choose buses as the mode of travel, while only 40% of high-income travelers choose to take buses, 50% of high-income travelers choose to drive PVs, and 10% choose to use SAVs. With the increase in bus fares, high-income travelers gradually give up on buses and choose SAVs with different levels of automation. When the bus fare is 1.5 RMB, some low-income travelers choose SAVs. Although the vast majority of them still choose to take the bus, the selection rate for SAVs with the lowest level of automation only slightly increases. As bus fares continue to rise, the number of travelers choosing to travel by SAV continues to increase. This means that, with the rise in bus fares, the advantages of SAVs are continuously improved. When the bus fare rises to 2 RMB, 137 low-income travelers choose SAVs, accounting for 17.125% of the total number of low-income travelers, while 50% of high-income travelers choose the SAVs with the highest automation level. Looking back at the entire fare increase process, we found that with the increase in bus fares, high-income travelers first experienced an increase in their choice of SAVs with the lowest level of automation, then decreased to 0. Finally, they turned their attention to SAVs with the highest level of automation. The choice rate for PVs has not changed, and the number of choices remains at 100. The range of choice rates for SAVs among low-income travelers has always been limited.

**Figure 1:**
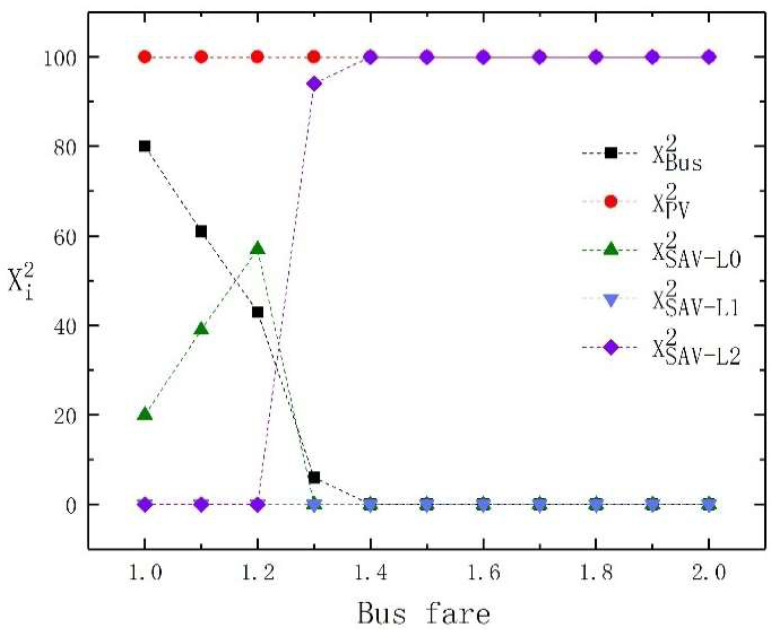
The travel mode change of high-income travelers with bus fare changes.

**Figure 2:**
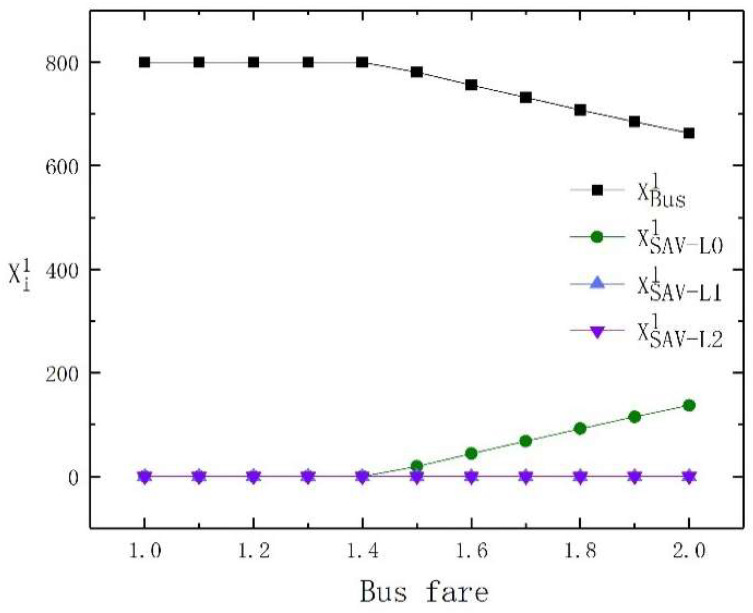
The travel mode change of low-income travelers with bus fare changes.

In order to further demonstrate the results, we show the changes in the travel mode choice of high-income travelers and low-income travelers under different bus fares in Figure 1 and Figure 2, respectively. From Figure 1 and Figure 2, it is obvious that both low-income and high-income travelers are attracted to buses when bus fares are low. As bus fares increase, high-income and low-income travelers turn to SAVs, but the high-income travelers choose SAVs with higher levels of automation, while low-income travelers choose SAVs with lower levels of automation.

In summary, we conclude that when bus fares are low, both low-income and high-income travelers are attracted to use buses. With the rise in bus fares, high-income and low-income travelers turn to SAVs, but high-income travelers choose SAVs with high automation levels, while low-income travelers choose SAVs with low automation levels.

#### 4.2.2. Sensitivity Analysis of Fuel Cost per Kilometer

**Figure 3:**
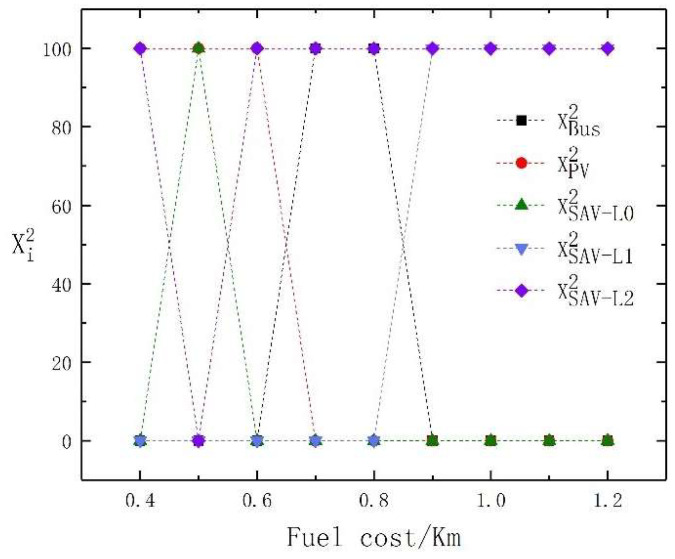
The travel mode change of high-income travelers with the change in fuel cost per kilometer.

**Figure 4:**
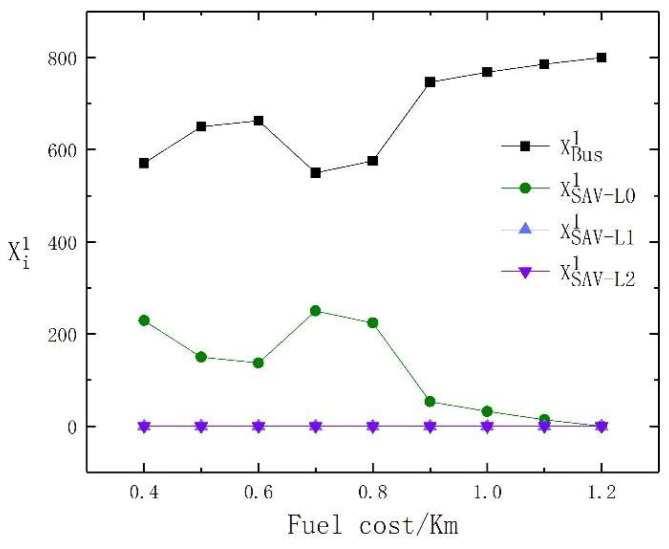
The travel mode change of low-income travelers with the change in fuel cost per kilometer.

The increase in fuel cost per kilometer directly affects the travel cost per unit distance of PVs and SAVs. Therefore, in this section, the sensitivity of fuel cost per kilometer to the choice of traveler’s mode of transport is analyzed. We assume that the travel price per kilometer of an SAV is 0.4 RMB higher than the fuel cost per kilometer. The fuel cost per kilometer ranges from 0.4 RMB to 1.2 RMB. The traveler’s mode choice is shown in Table 6. The changes in the travel mode choice of high- and low-income travelers under different fuel costs per kilometer are shown in Figure 3 and Figure 4, respectively. From Figure 3 and Figure 4, it is evident that when the fuel cost is low, some low-income travelers are attracted to SAVs with the lowest level of automation, while high-income travelers tend to prefer PVs. With the increase in fuel costs, low-income travelers all choose buses, while high-income travelers tend to prefer SAVs with different levels of automation.

It can be observed from Table 6 that when the fuel cost per kilometer is low, 229 low-income travelers choose SAVs with the lowest level of automation, while 100 high-income travelers choose SAVs with the highest level of automation. When the fuel cost per kilometer fluctuates in the range of 0.4 RMB to 0.6 RMB, the choice of SAVs among low-income travelers shows a downward trend, which also aligns with our impression that low-income travelers are more concerned about cost. As the utility of PVs is the highest, the proportion of PVs selected does not change, but there are fluctuations in the selection of SAVs with the lowest and highest levels of automation. Further comparing the results of high-income travelers’ selection of SAV automation levels, we find that high-income travelers switch from the selection of SAVs with the highest automation level to SAVs with the lowest levels of automation, and then the reverse. This is due to the mutual influence between distance-related variable costs and fixed costs.

When the fuel cost per kilometer reaches 0.6 RMB, the distance-dependent variable utility is higher than the fixed utility. At this time, for high-income travelers, the time cost will dominate. As the cost of fuel per unit kilometer continues to rise to 0.7 RMB, the advantages of PVs are weakened. High-income travelers no longer choose PVs. When the fuel cost per kilometer continues to rise to 0.9 RMB, due to the high travel cost of PVs and SAVs, more travelers choose to travel by bus. At this time, due to high congestion on the buses, some high-income travelers will abandon bus travel and choose SAVs.

For low-income travelers, there are only two modes of travel: buses and SAVs, but we assume that bus fares do not change with rising fuel costs per kilometer. When the fuel cost per kilometer fluctuates in the range of 0.4 RMB to 0.7 RMB, there is a downward trend in the choice of SAVs among low-income travelers. At the same time, as the utility of PVs is the highest, the proportion of PVs chosen by high-income travelers does not change, but there are fluctuations in the selection of SAVs with the lowest and highest levels of automation.

Further comparing the results of high-income travelers choosing the level of SAV automation, high-income travelers show a significant increase in using SAVs and then gradually decrease because high-income travelers are not sensitive to bus congestion, even when the bus is highly crowded, high-income traveler’s choice of travel mode does not change from buses to SAVs.

In summary, we conclude that as fuel costs increase, low-income travelers are more likely to choose buses, while high-income travelers choose higher-level SAVs. However, when both are low, there will be some differences in the choice of SAVs between high- and low-income travelers.

#### 4.2.3. Sensitivity Analysis of SAV’s Fee per Kilometer

**Figure 5:**
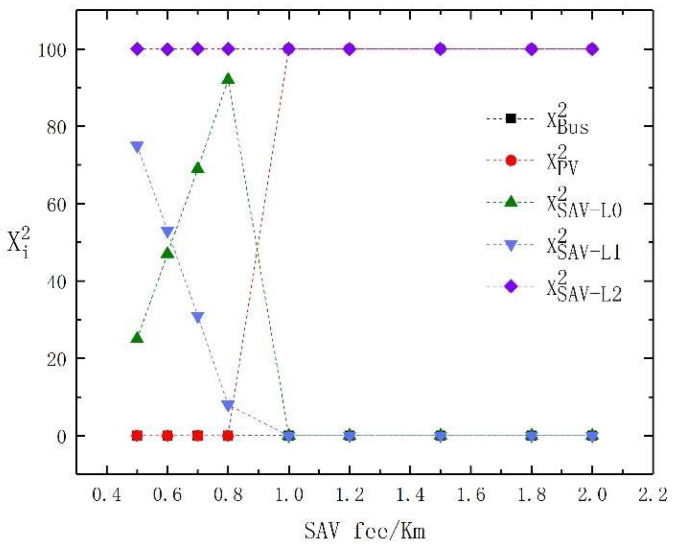
The travel mode change of high-income travelers with the change in SAV fees per kilometer.

**Figure 6:**
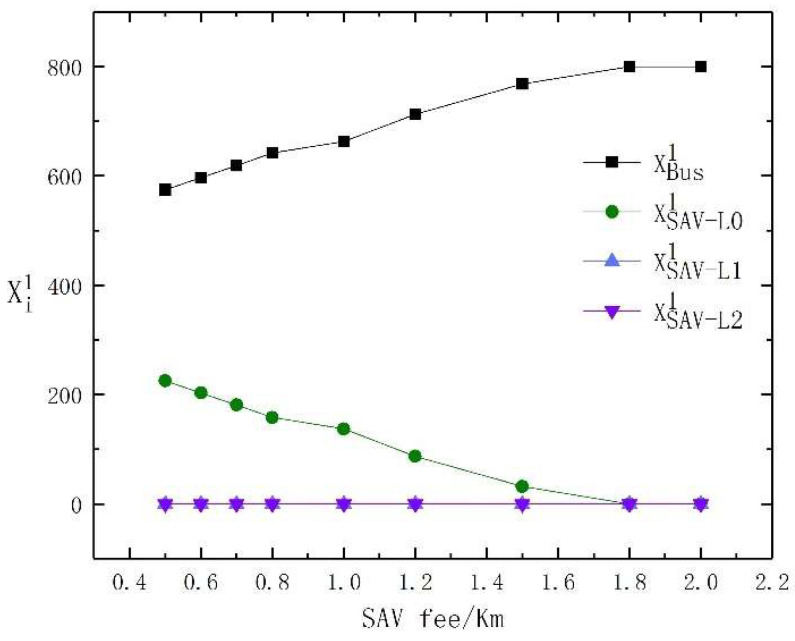
The travel mode change of low-income travelers with the change in SAV fees per kilometer.

The fee per kilometer of SAVs directly affects whether travelers choose them. We assume that the bus fare and the cost of PVs do not change, and further study the changes in the choice of travel modes for travelers within the fare per kilometer of SAVs range of 0.5 to 2 RMB. The test results are shown in Table 7. Clearer results regarding the changes in the travel mode choice of high- and low-income travelers under different SAV fees per kilometer are shown in Figure 5 and Figure 6. From Figure 5 and Figure 6, it is evident that when the SAV’s fee is low, some low-income travelers are attracted to SAVs with the lowest level of automation, while high-income travelers tend to prefer SAVs with different levels of automation. With the increase in SAV fees, all low-income travelers choose buses, while some high-income travelers begin to choose PVs.

For low-income travelers, when the fee per kilometer of SAVs is low, the utility of SAVs with low automation levels is similar to that of buses. Therefore, low-income traveler’s choice of travel mode fluctuates between buses and SAVs with low automation levels. However, highly automated SAVs still do not attract low-income travelers. As the fee per kilometer of SAVs rises, more low-income travelers choose to travel by bus. When the fee per kilometer of SAVs is 1.8 RMB, all low-income travelers choose to travel by bus.

For high-income travelers, when the fee per kilometer of SAVs, PVs, and SAVs are more effective than buses, travelers do not choose to travel by bus. When the fee per kilometer of SAVs is less than 1 RMB, parking fees are required when choosing PVs. Hence, all high-income travelers choose SAVs for travel. We further observe that when the fee per kilometer of SAVs is less than 0.7 RMB, high-income travelers tend to choose SAVs with a high level of automation, and as the fees increase, some high-income travelers choose SAVs with a low level of automation. When the fee per kilometer of SAVs is higher than 1 RMB, the travel utility of SAVs with low automation levels is lower than PVs, so SAVs with low automation levels are no longer selected, and the advantages of PVs and SAVs with high automation levels are more obvious.

In summary, we conclude that, as SAV fees increase, low-income travelers are more likely to choose buses, while high-income travelers choose higher-level SAVs. However, when both are low, there will be some differences in the choice of SAVs between high- and low-income travelers.

## 5. Conclusions

Due to the high costs of AVs, they are only owned by some large companies. Therefore, SAVs become the main way to popularize AVs. Due to their flexible, comfortable, and convenient features, SAVs have great advantages compared with PVs and buses, and so present strong competition. Therefore, the choice of three travel modes by heterogeneous users is studied with the aim of maximizing traveler utility, which is defined as a multiple-mode selection model with heterogeneous travelers. In the results testing section, the sensitivities of bus fares, fuel costs per kilometer, and SAV fees per kilometer are analyzed, and reasonable results are obtained. When bus fares are low, both low-income and high-income travelers are attracted by buses. With the rise in bus fares, high-income and low-income travelers turn to SAVs, but high-income travelers choose SAVs with high automation levels, while low-income travelers choose SAVs with low automation levels.

In the sensitivity analysis of fuel costs and SAV fees per kilometer, we obtain similar results. In other words, with an increase in the two parameters, low-income travelers are more likely to choose buses, while high-income travelers choose higher-level SAVs. However, when both are low, there will be some differences in the choice of SAVs between high- and low-income travelers. The level of automation of SAVs chosen by high-income travelers is higher than that of low-income travelers.

The model presented here is relatively simple. There are several ways in which it could be expanded. Based on the additional traffic induced by SAVs, the multiple mode selection models with unbalanced supply and demand should be studied in depth. The issue of competitive pricing between taxis and SAVs needs further discussion.

## Figures and Tables

**Table 1:** Contribution comparative table.

Author	Year	Contribution
Haboucha [14]	2017	The potential variables related to personal attitude and personal motivation for using autonomous vehicles are analyzed.
Fagnant [1]	2014	The design of an agent-based shared autonomous vehicle (SAV) operation model, the results of many case study applications using the model, and the environmental benefits of such settings compared with the ownership and use of traditional vehicles are described.
Vosooghi [15]	2019	Explores the impact of user trust and willingness to use SAEVs on the size of the Robo Taxi fleet.
Loeb [16]	2019	Estimates the cost of shared electric vehicles (SAEVs).
Sun. [17]	2020	Explores the internal mechanism of the elderly’s choice behavior in public transport, autonomous vehicles, and SAVs.
Kamel [18]	2019	Tries to take advantage of this granularity in order to explore the impact of user preferences on the modal split of shared autonomous vehicles.
Zhang [19]	2015	This study estimates the potential impact of an SAV system on urban parking demand under different system operation scenarios with the help of an agent-based simulation model.
Qin [20]	2023	This study designs a travel survey with cognitive experience for autonomous vehicles to obtain the stated preference data of different commuters for multimodal travel choices.
Bala [21]	2023	This article intends to advance future research about the travel behavior impacts of SAVs by identifying the characteristics of users who are likely to adopt SAV services and by eliciting a willingness to pay for service attributes.
Wu [22]	2023	In theory, this study contextualizes the trust-in-automation three-factor model, the UTAUT model, and trust theory and includes two domain-specific constructs (i.e., SAV anthropomorphism and SAV autonomy) to study public trust and acceptance towards SAVs.
Lee [23]	2019	This study investigates influential factors on the use of autonomous vehicles in terms of a technology acceptance model (which considers perceived ease of use, perceived usefulness, and intention to use) and factors for autonomous vehicle use (e.g., perceived risk, relative advantage, self-efficacy, and psychological ownership).
Shen [24]	2022	In order to investigate users’ intentions to use SAVs and the factors influencing them, a discrete choice experiment is designed derived from a Revealed Preference (RP) survey and a Stated Preference (SP) survey.
Krueger [25]	2017	This article intends to advance future research about the travel behavior impacts of SAVs, by identifying the characteristics of users who are likely to adopt SAV services and by eliciting a willingness to pay for service attributes.
Jing [26]	2019	This study is empirically tested using a valid survey sample collected from 906 respondents in China. The structural equation model was conducted to investigate the predictors of intentions to use AVs and SAVs.
Harper [27]	2016	This paper estimates bounds on the potential increases in travel in a fully automated vehicle environment due to an increase in mobility from the non-driving and senior populations, as well as people with travel-restrictive medical conditions.

**Table 2:** The parameters related to heterogeneous travelers.

	Low-Income Travelers	High-Income Travelers
α	0.5	0.4
D	12	12
VOTT	19.62	13.3

**Table 3:** The parameters related to travel modes.

	Buses	PVs	SAV-L0	SAV-L1	SAV-L2
$S_{i}$	19.2	24	24	24	24
$C_{i}$	2	2	0	0	0
$\lambda_{i}$	/	0.6	1	1	1
$T_{A}+T_{B}+T_{C}$	45.17	0	68.04	68.04	68.04
ω	2	/	2	2	2
ε	/	/	1	0.7	0.35
$Fare_{i}^{j}$	/	/	0	10	15
$\eta_{1}$	0.5	0.2	0.03	0.02	0.01
$\eta_{2}$	0.5	0.2	0.04	0.016	0.008

**Table 4:** The basic test results.

$X_{Bus}^{1}$	$X_{Bus}^{2}$	$X_{PV}^{2}$	$X_{SAV-L0}^{1}$	$X_{SAV-L1}^{1}$	$X_{SAV-L2}^{1}$	$X_{SAV-L0}^{2}$	$X_{SAV-L1}^{2}$	$X_{SAV-L2}^{2}$
663	0	100	137	0	0	0	0	100

**Table 5:** The test results of the sensitivity analysis of bus fares.

Bus Fare	2	1.9	1.8	1.7	1.6	1.5	1.4	1.3	1.2	1.1	1.0
$X_{Bus}^{1}$	663	685	708	732	756	781	800	800	800	800	800
$X_{Bus}^{2}$	0	0	0	0	0	0	0	6	43	61	80
$X_{PV}^{2}$	100	100	100	100	100	100	100	100	100	100	100
$X_{SAV-L0}^{1}$	137	115	92	68	44	19	0	0	0	0	0
$X_{SAV-L1}^{1}$	0	0	0	0	0	0	0	0	0	0	0
$X_{SAV-L2}^{1}$	0	0	0	0	0	0	0	0	0	0	0
$X_{SAV-L0}^{2}$	0	0	0	0	0	0	0	0	57	39	20
$X_{SAV-L1}^{2}$	0	0	0	0	0	0	0	0	0	0	0
$X_{SAV-L2}^{2}$	100	100	100	100	100	100	100	94	0	0	0
Total utility	20,859	19,926	18,957	17,950	16,907	15,827	14,714	13,618	12,484	11,278	10,054

**Table 6:** The test results of the sensitivity analysis of fuel cost per kilometer.

Fuel Cost/Km	0.4	0.5	0.6	0.7	0.8	0.9	1	1.1	1.2
$X_{Bus}^{1}$	571	650	663	550	576	747	768	786	800
$X_{Bus}^{2}$	0	0	0	100	100	0	0	0	0
$X_{PV}^{2}$	100	100	100	0	0	0	0	0	0
$X_{SAV-L0}^{1}$	229	150	137	250	224	53	32	14	0
$X_{SAV-L1}^{1}$	0	0	0	0	0	0	0	0	0
$X_{SAV-L2}^{1}$	0	0	0	0	0	0	0	0	0
$X_{SAV-L0}^{2}$	0	100	0	0	0	0	0	0	0
$X_{SAV-L1}^{2}$	0	0	0	0	0	100	100	100	100
$X_{SAV-L2}^{2}$	100	0	100	100	100	100	100	100	100
Total utility	19,743	20,046	20,859	21,996	22,793	23,265	23,724	24,122	24,469

**Table 7:** The test results of the sensitivity analysis of SAV fees per kilometer.

SAV’s Fee/KM	0.5	0.6	0.7	0.8	1.0	1.2	1.5	1.8	2.0
$X_{Bus}^{1}$	575	597	619	642	663	713	768	800	800
$X_{Bus}^{2}$	0	0	0	0	0	0	0	0	0
$X_{PV}^{2}$	0	0	0	0	100	100	100	100	100
$X_{SAV-L0}^{1}$	225	203	181	158	137	87	32	0	0
$X_{SAV-L1}^{1}$	0	0	0	0	0	0	0	0	0
$X_{SAV-L2}^{1}$	0	0	0	0	0	0	0	0	0
$X_{SAV-L0}^{2}$	25	47	69	92	0	0	0	0	0
$X_{SAV-L1}^{2}$	75	53	31	8	0	0	0	0	0
$X_{SAV-L2}^{2}$	100	100	100	100	100	100	100	100	100
Total utility	16,140	17,154	18,163	19,164	20,859	21,714	22,548	23,030	23,292

## Data Availability

Data sharing is not applicable to this article.
